# Exploring Autophagy in Drosophila

**DOI:** 10.3390/cells6030022

**Published:** 2017-07-12

**Authors:** Péter Lőrincz, Caroline Mauvezin, Gábor Juhász

**Affiliations:** 1 Department of Anatomy, Cell and Developmental Biology, Eötvös Loránd University, H-1117 Budapest, Hungary; concrete05@gmail.com; 2 Catalan Institute of Oncology-IDIBELL, Laboratory of Cancer Metabolism (LMC), Hospital Duran i Reynals, 08908 Barcelona, Spain; caroline.mauvezin@gmail.com; 3 Institute of Genetics, Biological Research Centre, Hungarian Academy of Sciences, H-6726 Szeged, Hungary

**Keywords:** Atg8a, autophagy, Drosophila, Ref(2)P/p62

## Abstract

Autophagy is a catabolic process in eukaryotic cells promoting bulk or selective degradation of cellular components within lysosomes. In recent decades, several model systems were utilized to dissect the molecular machinery of autophagy and to identify the impact of this cellular “self-eating” process on various physiological and pathological processes. Here we briefly discuss the advantages and limitations of using the fruit fly *Drosophila melanogaster*, a popular model in cell and developmental biology, to apprehend the main pathway of autophagy in a complete animal.

## 1. Introduction

### 1.1. A Brief Overview of Autophagy

The main protein degradation pathways are proteasomal and lysosomal breakdown in eukaryotic cells. Based on how intracellular material reaches the lysosome, several autophagic routes can be distinguished. During macroautophagy (simply referred to as autophagy in this paper), large portions of cytoplasm including whole organelles are captured by a membrane cistern (phagophore or isolation membrane) into a double membrane autophagosome, which then fuses with components of the endo-lysosomal system. Alternatively, lysosomes or late endosomes are able to directly engulf small portions of cytoplasm or organelles by microautophagy. Individual proteins may also reach the lysosomal lumen via Lysosome-associated membrane protein 2A (Lamp-2A) with the assistance of Hsc70 during chaperone-mediated autophagy (CMA) in mammalian cells. Of note, Lamp2A-driven CMA likely does not occur in Drosophila in the lack of a homolog for this protein. In all scenarios, acidic lysosomal hydrolases degrade cargo material and the resulting monomers are recycled back to the cytosol to fuel biosynthesis and energy production [1]. Autophagy is required to maintain cellular homeostasis in response to stress such as nutrient starvation. Inhibition of autophagosome biogenesis results in accumulation of selective autophagic cargo including Ref(2)P/p62-positive aggregates of polyubiquitinated proteins that may be cytotoxic. Misregulation of autophagy is associated with many diseases such as cancer and neurodegeneration [1,2]. 

Factors involved in autophagosome formation were first isolated in Saccharomyces cerevisiae and are called Atg (Autophagy-related) proteins. [3]. Most of these proteins have orthologs in higher eukaryotes including *Drosophila melanogaster*, and their functions are highly conserved [4]. The evolutionarily conserved complexes of Atg proteins regulate the initiation and elongation of phagophores and the formation of autophagosomes [5,6]. Autophagosomes fuse with lysosomes with the help of a Rab small GTPase (Rab7), the tethering factor HOPS, and SNARE proteins in yeast [7]. Interestingly, there appear to be important differences in the mechanisms of autophagosome-lysosome fusion and consequently autophagic cargo degradation between yeast and animal cells. The HOPS tethering complex and Rab7 are also involved in *Drosophila* and mammals [8,9,10] while the SNAREs are not homologous [11,12], and autophagosome degradation in animal cells also depends on Rab2 [13,14]. In addition, molecular motors including dynein and kinesins are also involved in the transport of autophagic vesicles [15,16].

### 1.2. Genetics of Drosophila

*Drosophila melanogaster* is a small dipterid insect historically used in research laboratories because of its powerful genetics, and several breakthroughs were achieved using this animal model in cell and developmental biology studies [17]. Fruit flies have a relatively short reproduction cycle of about 10 days, and their maintenance is cheap: it requires very simple instruments and much less human resources compared to vertebrate organisms. [18]. *Drosophila* can be easily manipulated and several strategies are available to generate mutant or transgenic *Drosophila* lines. One of the most common tools are mobile genetic elements (transposons), and many of the available transposon insertion lines cause loss-of-function of the affected gene. Moreover, appropriately located transposons can be used to generate null alleles via imprecise excision [19]. Transposons can also serve as vehicles to insert transgenes into the genome [20]. Additionally, genetic manipulations commonly utilize FLP/FRT recombination, phiC31 integrase-mediated targeted insertion, and more recently, CRISPR/Cas9 mutagenesis [21,22,23]. 

**Figure 1:**
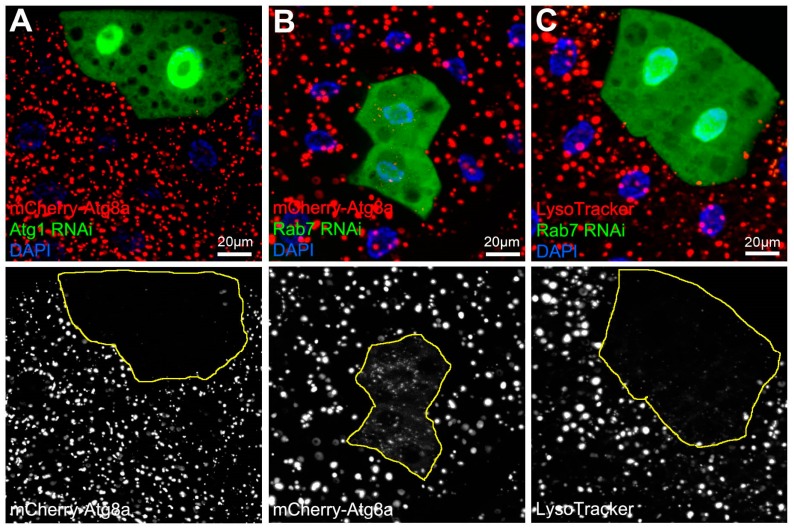

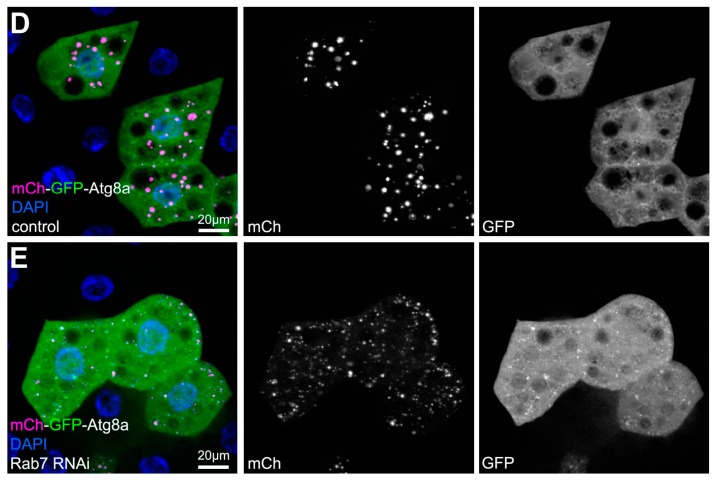
(**A**–**C**) Starved larval fat tissue samples from mosaic animals expressing RNAi constructs only in Green Fluorescent Protein (GFP)-positive cells. (**A**) GFP-marked Atg1 RNAi cells fail to induce autophagy as compared to surrounding control cells, based on lack of mCherry-Atg8a puncta. (**B**) As Rab7 is required for autophagosome-lysosome fusion, only faint and small mCherry-Atg8a dots (representing mostly autophagosomes) can be detected in GFP-positive cells depleted for Rab7, as opposed to the surrounding control cells where large bright structures (autolysosomes) are abundant. (**C**) LysoTracker staining also supports the detection of autolysosome formation seen in surrounding control cells, which is impaired in GFP-marked Rab7 RNAi cells. (**D**) The flux reporter mCherry-GFP-Atg8a shows that starvation-induced autophagic degradation proceeds normally in control cells, as GFP is quenched in autolysosomes while the mCherry signal remains prominent. (**E**) GFP fluorescence is retained and colocalizes with mCherry in Rab7-depleted cells, indicating a failure in autophagic flux. Note that mCherry-GFP double positive structures are also smaller than the structures seen in control cells, suggesting that these vesicles are most likely autophagosomes or small non-functioning autolysosomes.

Targeted gene expression is usually achieved with the UAS/Gal4 system. Many fly stocks carrying an Upstream Activating Sequence (UAS) in the promoter region of the gene of interest are available or can be easily generated. Gal4 drivers are used to express the gene of interest in selected cells or tissues, which is dictated by the promoter of the Gal4 line [24]. Somatic recombination is also a routine task, and it allows the comparison of genetically modified (mutant, knockdown or overexpression) cells to their wild type neighbors in the same tissue of the same mosaic animal (Figure 1A–C).

All these techniques can be combined to expand the range of experimental setups in different tests. This plasticity makes *Drosophila* ideal for large-scale RNAi or mutant screens [25]. The genome of Drosophila has been sequenced and the majority of its genes are conserved: approximately 75% of human disease genes can be found in flies [26]. Thus, research in flies can usually be translated to vertebrate organisms, including human.

### 1.3. Autophagy in Drosophila

*Drosophila* is a holometabolous insect, which means that the larval stages are followed by a non-motile life stage (first prepupa and then pupa) in which the whole body is reorganized. During this so-called complete metamorphosis, larval tissues undergo histolysis while diploid cells proliferate and differentiate to form the adult organs by the time the imago emerges from the pupal case. During fruit fly development, three subsequent larval stages (L1, L2 and L3) can be distinguished, interspersed with highly regulated transitions known as molting [18]. In mid-L3 larvae, an increase in the level of 20-hydroxyecdysone (the steroid molting hormone) at a low concentration of the sesquiterpenoid juvenile hormone triggers a behavioral change: larvae exit from the food and search for a suitable place for metamorphosis. During this phase of the wandering period, ecdysone-induced developmental autophagy is observed in most of the polyploid larval tissues including fat and intestine [27]. These cells use autophagy as a preparative phase of programmed cell death to free up stored biomass to be utilized by diploid cells as a nutrient and energy source during metamorphosis [27,28,29]. Autophagy likely plays an important role during metamorphosis, which is essentially a developmentally programmed 5-day starvation period. Developmental programmed autophagy is also observed during muscle remodeling in pupae, and it contributes to DNA fragmentation in nurse cells during oogenesis [14,30].

Autophagy also plays an important role in maintaining cellular homeostasis during basal conditions and in response to stresses such as nutrient scarcity. Despite what its name suggests, the insect fat body not only stores fat similar to human adipose tissue but it also plays an essential role in nutrient storage and utilization and has major metabolic functions. Thus, it can be considered as a liver-like organ of the insects [31]. Being the main storage site for lipids, glycogen and considerable amounts of proteins, fat body responds well and fast to nutrient starvation and swiftly releases lipids, carbohydrates and amino acids into the hemolymph (blood) [31]. In the laboratory, early L3 stage larvae are routinely exposed to a 20% sucrose solution to trigger amino acid starvation-induced autophagy in fat body cells. The advantage of this method compared to placing larvae in water is that the density of this solution is quite high, so it allows the larvae to float on the surface without drowning and suffocating. Autophagy is induced within 60–90 min with the maximum number of autophagic structures peaking at around 3–5 h in the fat body, and it provides nutrients such as amino acids to other organs [32]. Interestingly, exposing larvae to sucrose solution also induces large-scale synthesis of glycogen in fat cells [33].

The fat body develops from the embryonic mesoderm and consists of two lobes of cells organized in monolayers, which permits easy examination under the microscope. Thanks to these advantages, fat cells are often used to study autophagy in Drosophila. Other organs such as the compound eye are often used for the analysis of autophagy function in neurodegenerative disorders [34], and the larval salivary gland and intestine are excellent model tissues to study developmental autophagic cell shrinkage and death [35,36]. Larval midgut cells also respond well to starvation-induced autophagy, so this organ may offer a good alternative to fat body, especially for intracellular trafficking studies when epithelial cell polarization is of interest. Another tissue that was already successfully used to analyze starvation-induced autophagy in Drosophila is the ovary of adult females [37].

Analysis of the first autophagy gene null animal—an Atg7 deleted mutant—revealed a striking phenotype: despite strongly impaired starvation-induced and decreased developmental autophagy, adult flies developed with some delay but displayed no obvious morphological defects. Importantly, these flies were hypersensitive to starvation, oxidative stress, and had a short lifespan [38]. These phenotypes were also observed in the case of null mutants of other genes (Atg5, Atg16) that are required for the lipidation of Atg8a (the fly homolog of LC3 family proteins) [39,40]. All of the Atg8a lipidation system and Atg8a mutant flies are viable, probably because residual autophagic degradation remains in these animals as shown in mammalian cells [41]. Taken together, *Drosophila melanogaster* is an excellent tool to examine the role of autophagy during stress tolerance and aging.

While selective autophagy of ubiqutinated proteins is well established in Drosophila, the selective degradation of organelles has rarely been studied yet. A proteomics-based approach revealed that while mitochondria are degraded in lysosomes through the main autophagy pathway, respiratory chain subunits are selectively turned over independent of Atg7 [42]. This is possibly achieved by the formation of mitochondria-derived vesicles that can directly fuse with lysosomes in a Syntaxin 17-dependent manner, but this pathway is still unexplored in Drosophila [43,44].

It is important to highlight that using a complete animal to study autophagy has numerous advantages compared to cultured cells. On one hand, phenotypes can be evaluated on the organismal level, for example neuromuscular function can be tested in negative geotaxis (climbing) assays. On the other hand, physiological and pathological communication between different cells and tissues utilizing hormones and metabolites can be studied to understand the complex and often tissue-specific regulation of this process [45,46,47].

## 2. Advantages and Limitations of Commonly Used Autophagy Assay Methods in Drosophila

### 2.1. Electron Microscopy

Transmission electron microscopy (EM) is the classical method of visualizing and clearly distinguishing autophagic vesicles (Table 1). Of course, validation of EM results using confocal microscopy and biochemical assays is mandatory [48]. As autophagic structures in fly cells looks very similar to ones found in mammalian cells, researchers with previous EM experience on other model organisms would easily identify autophagic vesicles.

Fly tissues are usually dissected in ice cold PBS and then fixed with cold (4 °C) aldehyde fixative solution (for example, 2 to 3.2% formaldehyde with 0.5 to 1% glutaraldehyde, 1% sucrose and 0.028% CaCl_2_ in 0.1 N sodium cacodylate solution, pH 7.4 for overnight). Sucrose ensures optimal osmolality and calcium helps to preserve membranes. Fixed samples may be stored for a few days in fixative at 4 °C before embedding, which usually takes two consecutive days. To maximize the preservation of ultrastructure, post-fixation of samples with 0.5% osmium tetroxide for 1 h then with half-saturated aqueous uranyl acetate for 30 min at room temperature are usually carried out. These reagents also give good contrast to membranes and organelles. Then samples can be dehydrated in a graded series of ethanol and embedded into an epoxy resin according to the manufacturers’ recommendations. Hardening of this resin usually requires two days.

For fat body samples, carcasses are inverted, fixed and embedded. After the hardening step, fat body can still be recognized and unnecessary tissues and resin is easily trimmed off. We usually dissect the other organs (such as brain, salivary gland or midgut) before embedding. Immuno-EM (Table 1) can also be carried out using standard procedures, but in order to preserve the antigens it is often necessary to use acrylic resins (such as LR White) instead of epoxy ones and milder chemical fixation [12]. In some cases, the embedding method with progressive lowering temperature may be helpful to improve antigen preservation [49]. Of course, sucrose infiltration of fixed samples and cryo-ultrasectioning also works very well for immunogold labeling. A good alternative to these methods is correlative light and electron microscopy. Although this technique requires special equipment and experience, it would be very well-suited for the analysis of autophagy in Drosophila.

The ultrastructural analysis of adult brains permits detection of the accumulation of p62-positive large protein aggregates, which are a hallmark of impaired basal autophagy in neurons (Table 1) [50,51,52].

A nearly forgotten technique to unambiguously identify lysosomes and autolysosomes in ultrastructural sections is acid phosphatase enzyme cytochemistry. This classical method has been widely used more than half a century ago for the discovery and analysis of lysosomes in various organisms.. It is very simple to carry out in Drosophila: incubation of chemically fixed tissue samples in a substrate solution results in deposition of an electron dense precipitate in (auto)lysosomes containing acid phosphatase (Table 1) [13,49].

### 2.2. Confocal Microscopy

Although EM gives the most detailed information about organelle ultrastructure, it must be accompanied by other tests for the analysis of autophagy and flux. Also, it is not suitable for high-throughput studies such as genetic screens. Confocal microscopy is probably the most widely used technique to analyze autophagy in Drosophila. Staining with vital dyes as well as using fluorescent reporters to label autophagic vesicles are standard methods in flies. Most reagents used for fly samples are identical or very similar to the ones used in mammalian systems [53].

#### 2.2.1. Fluorescent Reporters

The most commonly used marker for autophagic vesicles is Atg8a, the fly ortholog of mammalian LC3 and yeast Atg8. Drosophila lines expressing GFP and/or mCherry-tagged-Atg8a (or even human LC3) are available from public stock centers and are suitable to examine both starvation-induced and developmental autophagy [27,32].

During autophagosome formation, Atg8a becomes covalently conjugated to phosphatidyl-ethanolamine and is bound to phagophore and autophagosome membranes. This is facilitated by ubiquitination-like protein conjugation systems that involve Atg7 (E1-like), Atg3 (E2-like) and a complex of Atg12-Atg5-Atg16 (E3-like). The advantage of Atg8a versus other Atg proteins is that high levels of lipidated Atg8a remain associated with completed autophagosomes [5]. Atg8a is bound to both the inner and outer membrane of the autophagosome. The cysteine protease Atg4 cleaves Atg8 from the outer membrane at a late stage of autophagosome maturation, while it remains associated to the inner membrane. Atg8a-positive vesicles accumulate inside autolysosomes in v-ATPase deficient cells in which lysosomal acidification is defective [54].

It is important to mention that most reporters (especially when overexpressed) can be degraded by autophagy in lysosomes, and RFP and its derivatives are surprisingly stable and accumulate to high levels within autolysosomes. This artefact complicates the interpretation of the results, so we suggest the use of complementary analyses to categorize autophagic vesicles. Lysosomes are often identified using a fluorescent-tagged lysosome-associated membrane protein (Lamp). These reporters label lysosomes independent from their acidification state and are very useful for the analysis of autophagic and endosomal degradation as well as biosynthetic transport to lysosomes (Table 1) [10,55]. Autolysosomes label very strongly with the mCherry-Atg8a reporter, because it is selectively targeted to the autophagosome and the intraluminal pool is transported to the lysosome, where due to its stability mCherry remains fluorescent. Thus, this reporter is generally used to label all autophagic structures including autophagosomes and autolysosomes (Table 1), and no punctate signal is detected when autophagosome formation is impaired (Figure 1A). It can even be used as a preliminary indicator of autophagosome fusion/maturation defects [50]. In this case, starved mutant cells accumulate small faint dots (autophagosomes) in contrast to control cells, which contain large, bright autolysosomes (Figure 1B) [8,12,13]. 

As overexpression of a reporter itself may bias the phenotypes, we recently switched to using triple-mCherry tagged Atg8a (3xmCherry-Atg8a) and Lamp reporters expressed from the Atg8a and Lamp genomic promoters, respectively (Table 1) [10,13,45]. Although expression from the genomic promoters is about an order of magnitude lower than that observed in case of overexpressions by Gal4/UAS or fat body-specific R4 promoters, the triple fluorescent tag makes it possible to easily visualize autophagic vesicles or lysosomes. Of note, these genomic promoter-driven triple-mCherry reporters work well not only for fat body experiments but also in other tissues such as larval wing disc or midgut cells [13,45]. 

Fluorescent or HA-tagged reporters detecting the selective autophagy cargo protein Ref(2)P/p62 are also available [10,56,57]. Ref(2)P/p62, the selective receptor of ubiquitinated proteins, is itself an autophagic cargo and is a subject of autophagic degradation [51,58]. Hence, the levels of p62 and autophagic degradation are inversely proportional. The accumulation of p62 aggregates in autophagy defective cells can be routinely monitored using confocal microscopy (Table 1) [10,57,59].

#### 2.2.2. Vital Dyes

The most commonly used dyes to stain larval fat cells are Lysotracker, and more recently, Magic Red (Table 1) [10,32,60,61]. These dyes are membrane permeable and accumulate in acidic, degradative organelles. In well-fed fat body cells, very few acidic vesicles are detectable, in contrast with starved cells. Under starvation-induced autophagy, the appearance of Lysotracker-positive vesicles marks a huge increase in the autolysosome compartment [32,61]. Magic Red-positive vesicles mark functional lysosomes/autolysosomes containing active cathepsin, a typical lysosomal hydrolase [10,54]. A cautionary note is that other cells such as macrophages and nephrocytes always contain large, Lysotracker-positive endolysosomes or phagolysosomes which could be misidentified as autolysosomes [62]. Thus, it is not possible to use these dyes as a proxy for autophagy in these cells.

#### 2.2.3. Antibodies

One disadvantage of using Drosophila is the scarcity of antibodies available to follow endogenous proteins by indirect immunofluorescence (IF) and western blots (WB), or to study protein interactions with immunoprecipitation assays at the endogenous level. Since Atg8a is the most common marker, antibodies against Atg8a have been generated in multiple laboratories and most of them work well in WB and IF microscopy experiments (Table 1) [37,40,52,63,64]. A commercially available antibody raised against human GABARAP can also be used in both applications for Drosophila samples (Table 1) [65]. Detection of endogenous Atg8a-positive vesicles is one of the best assays for identification of autophagosomes. An antibody against the SNARE Syntaxin17 (Syx17), which is required for autophagosome-lysosome fusion, is also available. Despite its autophagosomal localization [12], anti-Syx17 cannot be considered a specific autophagosome marker on its own, as Syx17 is also found on other organelles such as the ER and mitochondria (Table 1) [11]. Commercial antibodies against Drosophila Atg5 or Atg12 have also been developed and may be used to follow autophagy initiation as phagophore markers in IF experiments (Table 1) [12,66,67].

Two different anti-Ref(2)P/p62 antibodies have been described, which work well both in WB or IF experiments (Table 1) [51,59]. The handling of fly tissues does not require special equipment and most IF methods are carried out in a very similar way to vertebrate samples. For fat bodies, we find it most convenient to invert carcasses, and fix and stain using small baskets fabricated from Eppendorf-tubes immersed in 24-well plates. Fat bodies are usually dissected and mounted after the staining procedure. Other organs often require dissection before staining to make sure that they are properly exposed to solutions.

Interconnection of autophagy with other intracellular vesicular transport pathways can be analyzed by the detection of endosomes, the Golgi apparatus and ER markers for example. For this, an excellent antibody toolkit was generated in Sean Munro’s lab, and these reagents can be purchased from the Developmental Studies Hybridoma Bank [68]. Additionally, transgenic flies expressing fluorescently tagged reporters for these organelles can be used for the characterization of autophagy progression and mutant phenotypes. Such reagents were used to examine the properties of retromer-depleted cells. In those cells, the loading of lysosomes with lysosomal hydrolases is perturbed, that is why these cells accumulate enlarged acidic autolysosomes. These could have been falsely interpreted as increased autophagic activity, but EM analyses also revealed intact cytoplasmic material in these vesicles [69].

### 2.3. Western Blot (WB)

Western blots are usually carried out using samples prepared from whole animals or dissected tissues by boiling the collected samples in SDS-containing Laemmli buffer for 3 min, which is followed by homogenization. Then the boiling is repeated to recover as much protein as possible, followed by two centrifugation steps to get rid of debris and fat.

Autophagy is inhibited by TOR, which is a master regulatory kinase and signaling hub in eukaryotes. TOR activity can be analyzed by detection of phosphorylation levels of direct TOR targets such as S6K and 4EBP1. Commercial antibodies are available for this purpose (Table 1) [46,54,70]. These experiments can provide important insight into the regulation of autophagy induction in a given setting.

The main protein routinely detected in WB experiments is Atg8a (Table 1), which is carried out similarly to mammalian LC3 blots [53] and exploits the fact that the autophagosome-associated form of Atg8a (Atg8a-II) migrates faster than the non-lipidated form (Atg8a-I) during gel electrophoresis. Efficient separation of these two bands using at least 13% separating polyacrylamide gels helps assessing autophagy. An increase in Atg8a-II protein levels (relative to a loading control such as actin or tubulin) may indicate increased autophagosome numbers as seen in animals with autophagosome-lysosome fusion defects [8,10,12,13]. A decrease of Atg8a-II protein levels may indicates a defect in autophagosome induction and/or in Atg8a lipidation [50]. However, WB results should be interpreted with caution as in some Atg mutants the lipidated form of Atg8a accumulates to high levels [50], and an increase may as well indicate elevated autophagic activity. Thus, the interpretation of Atg8a immunoblots should be always carried out in parallel to flux (discussed below) and morphological assays to avoid misinterpretation of data.

## 3. Essential Concept of Approaches

### 3.1. Autophagic Flux

#### 3.1.1. Autophagic Flux Analysis by Microscopy

To follow autophagic degradation (flux) in flies, the tandem tagged mCherry-GFP-Atg8a reporters are routinely used (Table 1) [12,30,71] which work in a similar manner to mammalian RFP-GFP-LC3B reporters [53]. Briefly, the low lysosomal pH rapidly quenches GFP signal after autophagosome-lysosome fusion, thus autophagosomes appear as small dots positive for both GFP and mCherry, whereas autolysosomes are only positive for mCherry. Enlarged yellow structures can represent autolysosomes that fail to degrade internal material [8,12,50], and large-scale accumulation and clustering of small autophagosomes can also make it impossible to resolve individual vesicles by confocal microscopy. In such cases, ultrastructural analysis is again very useful to distinguish an autophagosome-lysosome fusion defect from impaired autolysosomal degradation.

Another standard assay for autophagic flux is to look at the intracellular accumulation of protein aggregates positive for Ref(2)P/p62 and ubiquitinated proteins (Table 1) [50,51,59]. To circumvent problems associated with Gal4/UAS mediated overexpression of p62 and transcriptional regulation of the endogenous gene product, we have recently generated a tubulin promoter-driven GFP-p62 reporter. This reporter is expressed at a constant low level in polyploid larval tissues and its protein level is mainly influenced by autophagic degradation, which proved to be a very sensitive measure of impaired flux [10].

#### 3.1.2. Autophagic Flux Analysis by Western Blot

In order to follow autophagic degradation in western blot experiments, Ref(2)P/p62 antibodies are commonly used and the increased amount of p62 often indicates the block of autophagic degradation (Table 1) [51,59]. Of note, the size of the Drosophila protein appears larger than its mammalian form (100 kD versus 62 kD). Other WB-based methods for the monitoring of autophagic flux are also available, which are mostly based on the autophagy-dependent intralysosomal conversion of tagged Atg8a reporters into free GFP or mCherry (Table 1) [50,72].

### 3.2. Treatment with Autophagy-Modulating Drugs

Both larvae and adults can be simply fed with various compounds such as rapamycin to modulate autophagy (Table 1) [32]. *Drosophila* is also suitable for medium-throughput drug testing using various disease models including neurodegeneration and high fat diet induced obesity and heart dysfunction. The polyamine spermidine is a good example for an autophagy-inducing compound that has proved to be effective for lifespan extension in various models including flies, and protected from toxicity induced by expression of Parkinson disease mutant alpha-synuclein or the Parkinsonian toxin paraquat (Table 1) [73,74,75]. Ecdysone analogs (such as RH 5849) can also effectively trigger autophagy (Table 1) [27]. Feeding larvae with Chloroquine (CQ) containing food is an effective method to block autolysosome acidification and induce myopathy (Table 1) [76]. Bafilomycin A1 may also be used to block autophagosome-lysosome fusion and acidification, but as TOR signaling can be affected under these circumstances, results should be interpreted with care (Table 1) [77]. Moreover, flies can be used to identify autophagy inducer drug candidates, such as AUTEN-67 (Table 1) [78].

Fly tissues can also be incubated with drugs ex vivo, which can be used to establish a direct effect in a given cell type and to avoid toxicity associated with administering certain drugs to whole animals. A good example for this strategy is Bafilomycin A1 [54].

## 4. Concluding Remarks

First, we would like to emphasize the importance of using multiple complementary assays to correctly estimate autophagy status in *Drosophila*, similar to other organisms. Second, the fruit fly model is particularly well-suited to study the basic mechanisms and regulation of autophagy, because its physiology and cell biology is surprisingly similar to that of humans, and established models exist to analyze various pathologies including cancer progression and neurodegeneration. There is still much room to investigate the various forms of autophagy in flies, for example selective organelle degradation and xenophagy. Future discoveries on the role and regulation of autophagy in Drosophila are expected to help understanding the importance of this process in humans.

## Figures and Tables

**Table 1:** List of methods discussed in this paper. See text for details.

Method		Practical Use
**Electron microscopy**	*Standard EM*	Identification of autophagic structures and protein aggregates on the ultrastructural level.
	*Immuno-EM*	Localization of proteins related to autophagy (such as Atg8a, Ref2P/p62, Syx17) on the ultrastructural level.
	*Acid phosphatase cytochemistry*	Detection of acid phosphatase to identify lysosomes on the ultrastuctural level.
**Confocal microscopy: *Reporters***	*GFP and/or mCherry-tagged-Atg8a*	Detection of autophagic structures (including autophagosomes and autolysosomes).
	*GFP and/or mCherry-tagged-Atg proteins (other than Atg8a/b)*	Detection of phagophores and autophagosomes. (Note: mCherry may accumulate in autolysosomes in these cases, too).
	*GFP and/or mCherry-tagged-Lamp proteins*	Detection of autophagosomes. (Note: Lamp proteins are not autolysosome specific markers.)
	*GFP-Lamp1 with mCherry-Atg8a*	To distinguish GFP-negative, mCherry-positive autophagosomes from autolysosomes and amphisomes, which are positive for both markers.
**Confocal microscopy: *Antibodies***	*anti-Atg5 or Atg12*	Detection of phagophores.
	*anti-Atg8a or anti-hGABARAP*	Detection of autophagosomes.
	*anti-Ref(2)P/p62*	Detects intracellular protein aggregates.
	*anti-Syx17*	Detects Syx17 positive structures. (Note: not all Syx17 positive structures are autophagosomes, Syx17 can be found on ER or mitochondria as well.)
**Confocal microscopy: *Vital dyes***	*LysoTracker, acridine orange*	Detection of lysosomes in most cells, while these are considered to be autolysosome specific in starved larval fat cells.
	*Magic Red*	Detection of functional lysosomes containing active cathepsins.
**Western blot**	*anti-Atg8a*	Detects cytosolic (non-lipidated, Atg8a-I) and autophagosome associated (lipidated, Atg8a-II) forms of Atg8a. The levels of the latter may correlate with autophagosome number or Atg8a lipidation.
	*anti-phospho-S6K and anti-phospho-4EBP*	Estimates TOR kinase activity, a main inhibitor of autophagy.
**Estimating autophagic flux**	*Tandem mCherry-GFP-Atg8a reporter (confocal microscopy)*	Estimation of autophagic flux. Functioning autolysosomes appear as mCherry+ dots, autophagosomes and non-functioning autolysosomes appear as mCherry+ GFP+ double positive dots.
	*Tagged Ref(2)P/p62 (confocal microscopy)*	Detection of intracellular protein aggregate accumulation, indicating impaired autophagic flux.
	*anti-Ref(2)P/p62 (western blot)*	The level of Ref(2)P/p62 is usually inversely proportional to autophagic degradation.
	*anti-GFP or mCherry (western blot)*	Conversion of GFP- or mCherry-tagged Atg8a reporters into free tags can be used to estimate autophagic flux. The levels of free GFP or mCherry is directly proportional to autophagic degradation.
**Drug treatments**	*Spermidine, ecdysone, rapamycin*	Feeding larvae with these chemicals induces autophagy.
	*Paraquat*	Feeding larvae with this Parkinsonian toxin results in oxidative stress induced autophagy.
	*Chloroquine (CQ)*	Feeding larvae with this compound inhibits acidification of lysosomes and induces myopathy.
	*Bafilomycin*	Inhibits autophagic degradation at multiple steps: both autophagosome-lysosome fusion and acidification are affected. (Note: this treatment may interfere with TOR signaling.)
	*AUTEN-67*	It is an autophagy inducing drug candidate.
